# Incidence trends of vestibular schwannomas in Denmark, Finland, Norway and Sweden in 1987–2007

**DOI:** 10.1038/bjc.2011.344

**Published:** 2011-09-06

**Authors:** S Larjavaara, M Feychting, R Sankila, C Johansen, L Klaeboe, J Schüz, A Auvinen

**Affiliations:** 1 Tampere School of Public Health, University of Tampere, Tampere 33014, Finland; 2 Karolinska Institute, Institute of Environmental Medicine, Stockholm 171 77, Sweden; 3 Finnish Cancer Registry, Pieni Roobertinkatu 9, Helsinki 00130, Finland; 4 Department of Cancer Epidemiology, Danish Cancer Society, Strandboulevarden 49, Copenhagen 2100, Denmark; 5 The Cancer Registry of Norway, PO Box 5313, Majorstuen, Oslo 0304, Norway; 6 STUK – Radiation and Nuclear Safety Authority, Laippatie 4, PO Box 14, Helsinki 00881, Finland

**Keywords:** neuroma, acoustic, incidence, registries

## Abstract

**Background:**

The reported incidence rates of vestibular schwannomas (VS) vary substantially, but it is unclear as to what extent the variation reflects differences in risk or recording practices. Our aim was to describe the incidence rates of VS in Denmark, Finland, Norway and Sweden between 1987 and 2007.

**Methods:**

Comprehensive data were available from all registries only for the period from 1987 to 2007. An analysis of a longer time period (1965–2007) was conducted with the Norwegian and Swedish data.

**Results:**

The average age-standardised incidence rates during 1987–2007 varied from 6.1 per 1 000 000 person-years (95% confidence interval (CI), 5.4–6.7) among Finnish men to 11.6 (95% CI, 10.4–12.7) in Danish men, and from 6.4 per 1 000 000 person-years (95% CI, 5.7–7.0) among Swedish women to 11.6 (95% CI, 10.5–12.8) among Danish women. An overall annual increase of 3.0% (95% CI 2.1–3.9) was observed when all countries and both sexes were combined, with considerable differences between countries. However, the practices of both reporting and coding VS cases varied markedly between countries and over time, which poses a challenge for interpretation of the results.

**Conclusion:**

The overall incidence of VS increased in all the four Nordic countries combined between 1987 and 2007, with marked differences between countries. However, the incidence rates more or less stabilised in the late 1990s, showing relatively constant incidence rates and even some decline after 2000.

Vestibular schwannomas (VS), or acoustic neuromas (or neurinomas), are benign intracranial tumours of the eighth cranial nerve. They develop from glial Schwann cells, which insulate neuronal axons in peripheral nerves in a manner similar to oligodendroglia in the brain. Schwannomas account for approximately 8% of all intracranial neoplasms (Louis *et al*, 2007). Vestibular schwannomas constitute approximately 60% of all schwannomas (Weller and Cervos-Navarro, 1977) and roughly 90% of intracranial schwannomas (Sarma *et al*, 2002; Propp *et al*, 2006).

The aetiology of VS is poorly known. Several risk factors have been proposed, such as radiation exposure in childhood (Schneider *et al*, 2008), loud noise (Edwards *et al*, 2006; Schlehofer *et al*, 2007; Hours *et al*, 2009), allergies (Schlehofer *et al*, 2007), epilepsy (Schoemaker *et al*, 2007), radiofrequency electromagnetic fields induced by long-term mobile phone use (Schoemaker *et al*, 2005; Ahlbom *et al*, 2009), high socioeconomic status (Schüz *et al*, 2010) and certain occupational exposures (Prochazka *et al*, 2010; Samkange-Zeeb *et al*, 2010). However, all these remain still tentative, owing to the lack of consistent evidence. The only well-established aetiological factor at present is neurofibromatosis 2 (Welling, 1998). Neurofibromatosis 2 accounts for <5% of all schwannoma cases (Louis *et al*, 2007), with carriers showing a 90–95% lifetime risk of VS, typically with multiple tumours (Asthagiri *et al*, 2009). The large majority of VS cases are, however, sporadic and of unknown aetiology (Louis *et al*, 2007).

The reported incidence rates of VS vary worldwide: from 1 to 20 cases per million inhabitants per year (Howitz *et al*, 2000; Tos *et al*, 2004; Gal *et al*, 2010; Stangerup *et al*, 2010). In addition to variation in risk between populations, this may reflect different classification systems with uncertain comparability between registries, as well as varying completeness of registration coverage.

The incidence rates of VS reported for various populations have been consistently increasing in the previous years (Stangerup *et al*, 2004; Tos *et al*, 2004; Propp *et al*, 2006; Stangerup *et al*, 2010). New diagnostic technologies (e.g., computerised tomography (CT) and magnetic resonance imaging (MRI)) or better awareness of both clinicians and symptomatic patients may have contributed to the increase. In addition, improved registration of brain neoplasms may have affected the reported incidence rates.

However, improvement in the coding systems may paradoxically decrease the number of recorded VS. As the classifications may have been inaccurate previously, schwannomas of other cranial nerve could not be distinguished from VS, which could inflate incidence rates (if interpreted as reflecting VS incidence). Thus, incidence could also be underestimated.

The aim of this study was to describe trends in incidence rates of VS in four Nordic countries between 1987 and 2007, and particularly to define temporal trends by country, sex and age group. The major advantages of the material include a long study period and large population of roughly 24 million people.

## Materials and methods

We identified all incident cases of VS notified to the Danish, Finnish, Norwegian and Swedish cancer registries from 1987 to 2007. The annual population sizes by 5-year age group and sex were obtained from the national population registries. Autopsy cases were also included (Curado *et al*, 2007).

In the Nordic countries, it is obligatory for all clinicians and pathological laboratories to notify all malignant and benign neoplasms of the brain and the central nervous system (CNS) to the national or regional cancer registries (Curado *et al*, 2007). The Danish Cancer Registry was founded in 1942, but the registration became compulsory by administrative order in 1987. In Finland, the reporting of cancer cases has been compulsory since 1961, in Norway since 1953 and in Sweden since 1958.

All the four countries had their own coding systems. In Norway and Sweden, the codes were based on various versions of the International Classification of Diseases (ICD) issued by the World Health Organisation, and the cancer registries in Norway and Sweden changed their classifications during the study period. However, in Denmark and Finland, national adaptations (adaptations based on ICD-codes) were used for VS, and these codes remained the same over the study period (Table 1). In all the countries, except in Finland, codes for malignant tumours were adopted (for historical reasons not known to the authors), despite the benign nature of VS. The national coding systems are presented in further detail in Table 1.

In Denmark, a Danish adaptation of ICD-7 was utilised (Table 1). The coverage was not fully comprehensive before the year 1987, when the notification of malignant tumours as well as all brain and CNS tumours became obligatory in Denmark (compulsory by administrative order) (Curado *et al*, 2007). Incompleteness of the Danish VS data before 1987 has been reported in a previous study (Howitz *et al*, 2000). The main study period was chosen to be from 1987 to 2007 owing to the limitations of the Danish data in the early years (1965–1986).

The Finnish Cancer Registry had its own coding system modified from ICD-7 and Manual of Tumor Nomenclature and Coding (MoTNaC) codes (Table 1). The Finnish data were comprehensive from 1979 onwards, but used from 1987 to 2007 (according to the common study period), with the exception of the analysis of age and birth cohort, in which data from 1979 to 1986 were also included (1979–2007).

In Norway, the coding systems applied from 1965 to 1992 were ICD-7 and MoTNaC. Later, from 1993 to 2007, the coding systems used were ICD-10 and MoTNaC (Table 1).

The coding system used in Sweden from 1965 to 1986 was ICD-7 supplemented by a PAD (pathologic–anatomic diagnosis) code. From 1987 to 1992, ICD-9 codes were utilised with SNOMED classification (Systematised Nomenclature of Medicine). However, as SNOMED codes were missing in many cases, PAD codes had to be used instead with ICD-9 codes. From 1993 to 2007, ICD-10 topography codes were used in combination with SNOMED codes (Table 1). In Sweden, the older coding was used in parallel with the newer coding, that is, the previous codes were recorded along with the newer codes when new coding practices were introduced. Therefore, as our primary interest was to evaluate the changes in incidence over time, for the period from 1987 to 2007, the coding ICD-9 together with PAD was used (even if more specific coding (ICD-10) was available from 1993 onwards).

For the longer study period from 1965 to 2007, analyses for incidence trends could only be performed for Norway and Sweden. For Norway, the changes in coding could not be overcome, and we had to use two separate coding systems for the period from 1965 to 1992 and 1993 to 2007 (Table 1). Whereas in Sweden, a similar coding had been applied throughout the entire period (along with the new coding systems) from 1965 to 2007. Thus, this older system (ICD-7 coding) was used in these analyses for Sweden.

The sensitivity analyses of the changes in classification were performed for Norway and Sweden. The analyses were carried out to evaluate the potential impact of the unspecified data on the incidence trends. Before 1993, the Norwegian data included all schwannomas of the cranial nerves, whereas since the introduction of ICD-10-coding, VS could be distinguished from other cranial schwannomas. Thus, assuming a stable ratio of non-VS and VS, we could quantify the extent of bias in incidence rates and trends due to unspecific coding before 1993. In Sweden, the older coding system (ICD-9) was used for the main analyses in 1993–2007, to provide consistency over the years. However, we compared the rates obtained by different coding systems, using the older and newer systems, and evaluated the impact of the different coding systems on the trends.

We calculated the incidence rates by 5-year age group and sex with age standardisation to the world standard population (Segi, 1960). The results were calculated separately for each country and combined for age-specific analyses. The confidence intervals (CIs) were calculated by verifying that the distribution followed the negative binomial distribution; the incidence rates of VS were expected to follow the negative binomial distribution.

The age groups for the age-specific analyses (0–44, 45–54, 55–64, 65 years and older) were formed aiming at similar numbers of cases in each group.

All data with complete coverage were combined (Denmark (1987–2007), Finland (1979–2007), Norway and Sweden (1965–2007)) in the analysis of age and birth cohort. This choice of accepting varying study periods, instead of only the common period of 1987–2007 for all countries, was reasoned to provide most information for the presentation.

For Norway and Sweden with longest period (1965–2007), we evaluated whether there is a difference in annual increase in incidence between the former and the latter part of the study period. The mid-point for this purpose was chosen to be the end of year 1986, thus forming two periods of approximately similar length from 1965 to 1986 and 1987 to 2007. The latter study period was equal to the main study period used in this study.

Negative binomial regression methods were used to estimate the average change over time. Likelihood ratio tests were applied to evaluate statistical significance of the interaction terms (nested within the main effects model) in the analyses of effect modification, that is, variation in incidence trends by country, age group and sex. The departure from linearity was assessed comparing year as a continuous variable to 3-year categories, also separately for each country.

Stata 8.2 (StataCorp, College Station, TX, USA) statistical software was utilised for all analyses.

## Results

A total of 5133 VS were registered during 1987–2007, of which 52% were in women (Table 2).

The average age-standardised incidence rates by country were estimated for a period of 21 years (1987–2007). The rates ranged from 6.1 per 1 000 000 among Finnish men to 11.6 in Danish men. For women, the lowest rates were 6.4 per 1 000 000 in Sweden and highest 11.6 in Denmark (Table 3).

**Figure 1:**
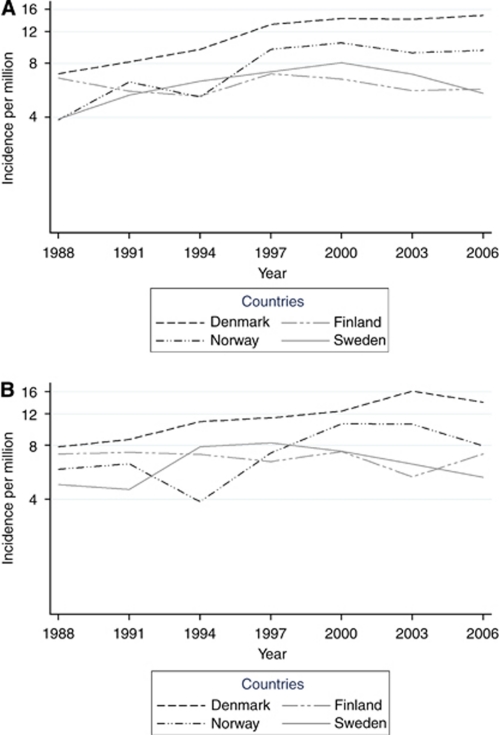
Age-standardised incidence rates (logarithmic scale) of VS by 3-year period, country and sex. (**A**) Men and (**B**) women.

An increasing trend was observed in 1987–2007, when all countries and both sexes were combined (3.0% per year, 95% CI, 2.1–3.9) (Figure 1A and B). The overall annual increase, estimated using negative binomial regression, was 3.5% (95% CI, 2.2–4.7) for men and 2.6% (95% CI, 1.4–3.9) for women. There was no interaction between period and country, and thus no indication of heterogeneity was present (*P*=0.96). The trends were comparable for women and men (*P*=0.36).

In country-specific analyses, the average annual increase ranged from a decrease of 0.2% in Finland (95% CI, −2.5 to +2.3) to 5.5% in Norway (95% CI, 2.8–8.3) for men, and for women from a decrease of 0.7% in Finland (95% CI, −2.8 to +1.4) to an increase of 4.7% in Norway (95% CI, 2.1–7.5). However, the increasing trend was not statistically significant in Finland nor Sweden in either sex, and the average trend was even decreasing (nonsignificantly) in both men and women in Finland (Table 3). There was no statistically significant departure from linearity in any country (*P*=0.97) (Figure 1A and B).

**Figure 2:**
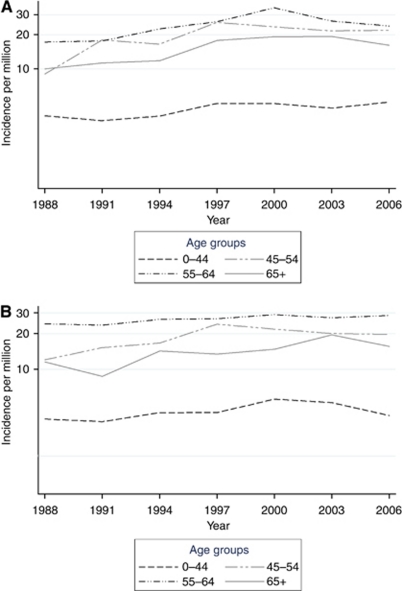
Age-specific incidence rates (logarithmic scale) of VS by 3-year period and sex. (**A**) Men and (**B**) women.

Incidence increased (with a lower confidence limit above zero) during the study period in all age groups, except in men aged 0–44 (increase of 1.8% (95% CI, −0.13 to +3.8)) and in women aged 0–44 (1.3% (95% CI, −0.60 to +3.2) and 55–64 years (1.3% (95% CI, −0.33 to +2.9)) (Figure 2A and B). The incidence trends did not show heterogeneity by age group in the analyses of effect modification (*P*=0.08). The annual average increase was highest in the age group 65 years or older in both sexes; 4.0% (95% CI, 1.8–6.3) for men and 3.9% (95% CI, 1.8–6.1) for women. When both sexes were combined, the overall increase was highest in age groups of over 65 years (4.0% (95% CI, 2.4–5.6)).

**Figure 3:**
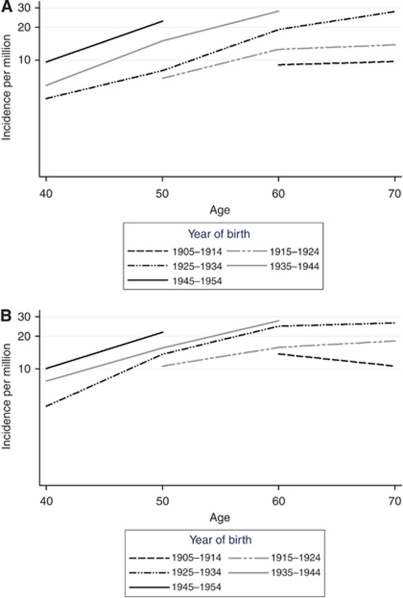
Cohort effect (incidence rates by logarithmic scale) of VS by age and sex. (**A**) Men and (**B**) women.

In an analysis by age and birth cohort from all the four countries, all data with complete coverage were combined. The results indicated a cohort effect with a higher incidence for later birth cohorts in practically all age groups (Figure 3A and B). An age effect was also present with increasing incidence by age, with the exception of the oldest birth cohort showing a decline after age 60 in women. The differences by birth cohort were more pronounced among men than women.

In the two countries with available data from 1965 to 2007, further analyses were conducted subdividing the study period into two segments, 1965–1986 and 1987–2007. In Norway, the average annual increase in the first period was 1.5% (95% CI, −0.58 to +3.5) and in the second period 5.0% (95% CI, 3.1–6.8). In Sweden, the annual increase in the first period was 3.4% (95% CI, 2.0–4.8) and in the second period −2.6% (95% CI, −4.9 to −0.34) (using ICD-7 193.0 through the entire period).

In Norway, when evaluating the proportion of VS from all intracranial schwannomas, 753 VS (code C72.4) were diagnosed during 1993–2007 for men and women combined, whereas during that time three schwannomas of the olfactory nerve (C72.2), one of the optic nerve (C72.3) and 56 schwannomas of the other and unspecified cranial nerve (C72.5) were diagnosed. Thus, the proportion of schwannomas arising from other cranial nerves was 0.5%, whereas those of unknown or unspecified site made up to 7% (*N*=56) of the total number of schwannomas (*N*=813) diagnosed in 1993–2007.

The main analyses for Norway for the period 1993–2007 were conducted with only confirmed VS cases. Thus, an assumption was made that the unspecified cases did not include any VS (i.e., cranial schwannomas of an unspecified site (C72.5) were not counted as VS). However, these unspecified schwannomas probably had relatively similar proportions of VS (93%) and other cranial nerves (0.5%), as those with a detailed diagnosis (missing at random). For the sensitivity analysis, the maximal and minimal incidence of VS was estimated by assuming that all the cases without a specific site were VS and none of the other cranial nerves, or *vice versa*. However, even such an extreme assumption had no effect on the increasing incidence trend in Norway for 1993–2007 (assuming that none of the unspecific schwannomas being VS, the annual increase for the study period was 5.5% (95% CI, 2.3–8.9), whereas assuming that all unspecific cases (C72.5) being VS, the annual increase was still 5.6% (95% CI, 2.5–8.8)). The age-standardised incidence rates for Norway for that period, assuming that none of the unspecified schwannomas was a VS, were 8.8 (95% CI, 7.9–9.6) per 1 000 000 for men and 8.1 (95% CI, 7.2–8.9) for women. However, if assuming that all the unspecified cases (C72.5) were VS, the rates were 9.4 (95% CI, 8.5–10.3) per 1 000 000 for men and 8.7 (95% CI, 7.9–9.6) for women.

In Sweden, in the main analyses for the period 1993–2007, the older more unspecific system (ICD-9 in combination with PAD) introduced in 1987 was used to provide consistency over time. Analyses on the impact of changes in coding systems in trends and incidence rates were carried out by comparing the trends obtained with the more unspecific system (older system, ICD-9+PAD), the coding used systematically for VS during that period in the Swedish Cancer Registry (ICD-10 (C72.4, C72.5, C72.9)+SNOMED) (see Table 1), and the most accurate and certain coding for VS (including only the ICD-10 code for VS (C72.4)+SNOMED).

The employment of the coding system for previous years (used from 1987 onwards; ICD-9 and SNOMED) rendered the number of VS to 1205 (total for both sexes, in 1993–2007), and the age-standardised incidence to 6.8 (95% CI, 6.3–7.3) per 1 000 000 for men and 7.0 (95% CI, 6.5–7.6) for women. However, with the systematically used coding in Sweden for that period 1993–2007 (using ICD-10 and SNOMED; see Table 1), the number was considerably (20%) higher (*N*=1452), with age-standardised rates of 8.3 (95% CI, 7.7–8.9) per 1 000 000 for men and 8.2 (95% CI, 7.7–8.8) for women. Yet, when only the most specific code for VS (C72.4, and not the unspecific codes C72.5 and C72.9) was included, the number of cases in 1993–2007 was substantially reduced (15%) (*N*=1026) and the age-standardised rates for men 5.7 (95% CI, 5.2–6.2) per 1 000 000 and 5.9 (95% CI, 5.4–6.4) for women was also reduced. Following the same order, the annual trends were −2.6% (95% CI, −4.9 to −0.34), −3.2% (95% CI, −5.3 to −1.0) and −2.8% (95% CI, −5.1 to −0.40), respectively.

## Discussion

This is the largest study carried out on incidence of VS with over 5000 cases spanning two decades. The average annual increase was 3.0% (95% CI, 2.1–3.9) in all countries combined. All countries showed some increase over time, except in Finland (increase ranging between the countries from −0.16 to 5.5% annually in men, and from −0.70 to 4.7% in women). However, the trends differed by age group and the patterns of increase varied across countries. Most of the increase occurred before the late 1990s. These could reflect either genuine increases in occurrence or improved diagnostics or reporting. The fall in registrations in the late 1990s may represent saturation in diagnosing prevalent cases that had not been diagnosed in the earlier years due to inaccurate diagnostic imaging methods (Nelson *et al*, 2006).

Denmark had the highest incidence rate throughout the entire study period, and in later years the difference between Denmark and the other countries even widened. The incidence rates in Finland remained relatively stable during the study period. For Norwegians, there was rapid increase in the incidence rates in the late 1990s for both sexes, but the increase levelled off in the 2000s (the numbers of new VS cases even decreased in both sexes). In Sweden, the rates increased somewhat more modestly until the end of the 1990s, when the numbers of incident VS cases started to gradually decrease.

Highest incidence rates were found in the age groups 45–54 and 55–64 years. This lower incidence among the elderly is highly unusual for a neoplastic disease and consistent with a detection bias due to lower diagnostic activity at old age. An increase over time was found at ages 65 years and older, but the older birth cohorts showed a decreasing or a stable rate after age 60 years.

Comparability of information is a major issue in any analysis of time trends over a long time period, as well as in international comparisons. Accurate case counts are dependent on both notification and classification. This is particularly important for benign tumours, which are typically not reported as exhaustively as malignant tumours (Curado *et al*, 2007). Improved diagnostic methods and awareness among the public and physicians may lead to an apparent increase in the VS incidence. Since the introduction of CT in the late 1970s and MRI in the 1980s, incidence rates of brain tumours, especially benign and slow growing, have increased. A recent study suggests that an indolent VS may be detectable in up to 0.03% of the population (Morris *et al*, 2009). These non-invasive and relatively inexpensive diagnostic methods have affected the detection of latent cases, particularly among the elderly (Inskip *et al*, 1995). In our study, the increase in incidence rates over time was seen mainly among the elderly (⩾65 years of age). Improved classification of brain neoplasms has certainly an impact on incidence rates, but the rates do not necessarily increase with improving classification as both overestimation and undercount may be reduced.

The coding protocols varied considerably between the countries. Not only did the coding vary internationally, but the classification schemes also changed in Norway and Sweden during the study period. This complicated the interpretation of the trends in these two countries, as VS could be distinguished from neurinomas at other locations only towards the end of the study period. However, this change in classification was partly overcome in Sweden, as the former coding systems remained in parallel, when a new coding system was introduced.

Denmark, with clearly the highest incidence rates of VS in the Nordic countries examined in our study, has a nationwide clinical database, which contains all histologically verified VS cases from all six neurosurgical departments that treat patients diagnosed with a VS in Denmark (Howitz *et al*, 2000). This nationwide clinical database does not exist in any other Nordic country, possibly explaining the higher rates in Denmark (with systematic reporting and registration for this type of tumour, as physicians are aware of an active nationwide clinical database).

The data were incomplete in Denmark and Finland during the early years, and therefore the statistical analyses could only be performed in the study period when all four countries had the most comprehensive data (from 1987 onwards).

Interestingly, the two countries with the longest study period showed different trends in 1965–1986 compared with 1987–2007. In Norway, the increase was more rapid in the latter period, whereas in Sweden there was a decrease in the incidence in the second period. The change in the coding protocol could not be overcome in Norway. However in Norway, despite the fact that the sensitivity analysis showed that the improved coding produced lower incidence rates than with the more ambiguous coding before 1993 (as we assumed in the calculations for 1993–2007 that none of the unspecified schwannomas was a VS (only ICD-10 code C72.4 was included in the counts)), a steep increase was observed, particularly during the earlier part of the latter period. For the years 1987–1992 with the ‘old coding’, the annual increase increased strongly in comparison to the earlier period (4.2%, 95% CI, −7.5 to +17.5), whereas for the years 1993–2006 the increase was high (5.5%, 95% CI, 2.2–8.9). However, the increase in Norway was sharp in the latter period only until the early 2000s, when it stabilised (and even decreased). This increase cannot be due to the changes in coding, as this improvement in coding should have, on the contrary, decreased the number of cases diagnosed as VS. Yet, there may be other issues influencing the incidence rates related to the new coding system that are not known to the authors (e.g., raising awareness among clinicians when introducing the new coding system, and improvements in case ascertainment). In Sweden, the coding remained the same, but the annual increase has slowly levelled off since the end of the 1990s, which could reflect the impact of the improved detection rate after the introduction of CT and MRI. In the late 1990s, these radiological imaging technologies were widely available in institutions responsible for diagnosing brain tumours in the Nordic countries.

The sensitivity analyses (number of cases including also the more unspecific coding compared to the cases with only the most accurate coding of the time) could be performed for Norway and Sweden. The sensitivity analyses were not possible in Denmark and Finland, as they did not change their coding over the years.

The annual trend in Norway in 1993–2007 was similar (5.5 and 5.6%) in both analyses, that is, assuming that none of the unspecified schwannomas was a VS, or *vice versa*. The age-standardised incidence rates were approximately 7% higher, assuming that all unspecified schwannomas were VS. However, this assumption is unlikely to be true, even if it is probable that most (but not all) of the unspecified schwannomas are VS. Thus, the incidence rates in Norway are likely to be approximately 5% (in the range 0–7%) higher than the main results as suggested in our study.

The interpretation of the results from the sensitivity analyses in Sweden was difficult, as even the most accurate coding (from 1993 to 2007) included non-VS (and there was no possibility to discriminate true VS from the cases with uncertain location). Nevertheless, we evaluated the rates in Sweden in 1993–2007 using three different classifications. The annual trends were relatively similar with all the three different inclusion criteria (ranging from −2.6% with the older coding system (ICD-9) to −3.2% with the system currently in use (ICD-10 including C72.5 and C72.9)). However, the age-standardised incidence rates in 1993–2007 were approximately 22% higher in men and 18% higher in women with the system currently in use in Sweden in comparison to the coding used in this study (ICD-9). Yet, there are no means to distinguish the true VS cases from the cases coded under C72.5 (other and unspecified cranial nerve) and C72.9 (for CNS, unspecified), which had to be included in these calculations as a substantial proportion of VS has been classified under these codes in previous Swedish VS studies (unpublished data). Using only the most specific VS code (C72.4), the crude incidence rates were, on the contrary, approximately 16% lower in both men and women in comparison to the coding system used for the rates in this study. Therefore, it is reasonable to assume that the rates presented in this study (with the older coding system, ICD-9) are rather realistic, with a potential margin of error of a maximum of one-fifth.

We used different study periods for different countries for the age and cohort analysis, which may have affected the rates in the analysis. If the diagnostic procedures for VS, patterns of development or behaviour of VS differ between countries, the cohort model may not represent reliably the changes over time owing to differences in study periods between countries. However, the longest complete study periods from all countries were chosen to provide the best representation of the influence of age and cohort in incidence of VS.

This study had several strengths. The study period was long (21 years for all countries with up to four decades for Norway and Sweden) with over 5000 VS cases. The population-based Nordic cancer registries are considered a benchmark in cancer registry quality. Notification legislations have remained consistent in all countries throughout the period. The registration is based on a unique personal identification number assigned to all inhabitants in the Nordic countries eliminating the possibility of duplicate registration. In addition, public health-care systems provide comprehensive coverage of the population, minimising differences in access to care, and in the quality of services.

In conclusion, our study shows an increase in VS incidence, mainly before mid-1990s. Increase occurred mostly in old (65+ years) population and the increase was relatively similar for men and women. The increasing trend was highest in Denmark (with initially the highest rates), whereas the increase was not seen in Finland, and was not pronounced in Sweden. These changes could be attributable to increasing risk or improvements in diagnostics and registration.

## Figures and Tables

**Table 1:** Diagnostic classification of vestibular schwannoma by period and country

**Denmark**	**Finland**	**Norway**	**Sweden^a^**
*1965–1986*	*1965–1978*	*1965–1992*	*1965–1986*
Incomplete coverage before 1987	Incomplete coverage before 1979	ICD-7 (193.1), MoTNaC (9560)	ICD-7 (193.0), PAD (451, 456)
			
*1987–2007*	*1979–2007*		*1987–1992*
National variation of ICD-7 (293.2)	National coding system (937)		ICD-9 (192.0), SNOMED (9560/0, 9560/3)
		*1993–2007*	*1993–2007*
		ICD-10 (C72.4), MoTNaC (9560/09, 9570/09)	ICD-10 (C72.4, C72.5, C72.9)^b^, SNOMED (9560/0, 9560/3; also 8000/0, 8000/3 with C72.5 or C72.9)

Abbreviations: ICD=International Classification of Diseases; MoTNaC=Manual of Tumor Nomenclature and Coding; PAD=pathologic–anatomic diagnosis; SNOMED= Systematised Nomenclature of Medicine; VS=vestibular schwannomas.

Denmark: A Danish adaptation of ICD-7 was utilised (a special code (293.2) assigned for VS). This code is derived from the code ICD-7 193 (malignant neoplasms of the brain and other parts of the nervous system) and the code 293.2 is reserved for tumours of the acoustic nerve.

Finland: A national coding was used with a topological code for vestibular nerve (937) used with further specification for benign behaviour (5) and histological type (code 45 for neurinoma). These codes were later converted automatically into ICD-O-3 codes for the years 1979–2007 (topography C72.9 for central nervous system, unspecified, and morphology M9560/0 for neurolemoma, benign).

Norway: 1965–1992 ICD-7 (193.1 for malignant neoplasm of the spinal cord, used systematically for schwannomas for unknown reason, covering schwannomas of all cranial nerves) and MoTNaC (code 9560 for schwannoma); 1993–2007 ICD-10 (C72.4 for neoplasm of the acoustic nerve) and MoTNaC (9560/09 for unspecified schwannoma and 9570/09 for neuroma of unspecified malignancy).

Sweden: The coding guidelines to the Swedish Cancer Registry are presented by each time period, but former coding systems are used in parallel with the newer systems throughout the registration.

1965–1986 ICD-7 (193.0 for malignant neoplasm of the brain, reason for the choice is uncertain) with PAD (451 for neuroma, 456 for malignant neuroma); 1987–1992 ICD-9 (192.0 for malignant neoplasm of cranial nerve) with SNOMED (9560/0 for neuroma, 9560/3 for malignant neuroma). However, as SNOMED codes were missing in many cases and PAD cases were available for everyone, PAD cases (451, 456) were used. The 1993–2007 ICD-10 (C72.4 for vestibular nerve, C72.5 for other and unspecified cranial nerve and C72.9 for central nervous system, unspecifed) with SNOMED (9560/0, 9560/3, and also 8000/0 for benign neoplasm and 8000/3 for malignant neoplasm with the codes C72.5 or C72.9).

In this study, we used for the main study period (1987–2007) ICD-9 (192.0) combined with PAD (451, 456), to provide consistency over time.

a The coding guidelines to the Swedish Cancer Registry are presented; however, former coding systems were used in parallel with the newer systems. In this study, we used for the main study period (1987–2007) ICD-9 (192.0) combined with PAD (451, 456), and for the total period (1965–2007) ICD-7 (193.0) with PAD (451, 456), to provide consistency.

b A substantial proportion of VS have been classified under the codes C72.5 (other and unspecified cranial nerve) and C72.9 (for central nervous system, unspecified) in previous Swedish VS studies (unpublished data).

**Table 2:** Number of VS for both sexes combined by 3-year period in Denmark, Finland, Norway and Sweden in 1987–2007

	**1987–1989**	**1990–1992**	**1993–1995**	**1996–1998**	**1999–2001**	**2002–2004**	**2005–2007**	**Total**
*Denmark*								
Men	69 (46)	82 (48)	98 (46)	138 (52)	156 (53)	159 (46)	173 (49)	875
Female	81 (52)	89 (52)	114 (54)	126 (48)	136 (47)	190 (54)	177 (51)	913
Total	150	171	212	264	292	349	350	1788
								
*Finland*								
Men	56 (44)	51 (42)	50 (42)	71 (50)	71 (47)	56 (50)	59 (44)	414
Female	70 (56)	70 (58)	69 (58)	70 (50)	79 (53)	57 (50)	74 (56)	489
Total	126	121	119	141	150	113	133	903
								
*Norway*								
Men	30 (38)	46 (50)	42 (55)	78 (55)	90 (49)	85 (46)	91 (54)	462
Female	50 (62)	46 (50)	34 (45)	63 (45)	93 (51)	101 (54)	76 (46)	463
Total	80	92	76	141	183	186	167	925
								
*Sweden*								
Men	66 (44)	86 (53)	104 (43)	129 (48)	139 (51)	118 (51)	95 (50)	737
Female	85 (56)	75 (47)	139 (57)	139 (52)	133 (49)	113 (49)	96 (50)	780
Total	151	161	243	268	272	231	191	1517

Abbreviations: VS=vestibular schwannomas.

Percentages of cases by sex are shown within the parentheses.

**Table 3:** Average age-standardised incidence rate per 1 000 000 person-years and average annual increase in percentages (with 95% confidence intervals) in 1987–2007

	**Incidence rate**	**Annual increase (%)**
*Denmark*		
Men	11.6 (10.4–12.7)	5.3 (2.7–7.9)
Women	11.6 (10.5–12.8)	4.5 (2.2–7.0)
		
*Finland*		
Men	6.1 (5.4–6.7)	−0.16 (−2.5, +2.3)
Women	6.9 (6.2–7.6)	−0.70 (−2.8, +1.4)
		
*Norway*		
Men	7.7 (6.9–8.5)	5.5 (2.8–8.3)
Women	7.5 (6.7–8.2)	4.7 (2.1–7.5)
		
*Sweden*		
Men	6.2 (5.6–6.8)	1.5 (−0.56, +3.6)
Women	6.4 (5.7–7.0)	0.44 (−1.7, +2.7)
